# Associations of granulocyte colony-stimulating factor with toxicities and efficacy of chimeric antigen receptor T-cell therapy in relapsed or refractory B-cell acute lymphoblastic leukemia

**DOI:** 10.1007/s00262-024-03661-1

**Published:** 2024-04-17

**Authors:** Sha Ma, Ying Wang, Kunming Qi, Wenyi Lu, Yuekun Qi, Jiang Cao, Mingshan Niu, Depeng Li, Wei Sang, Zhiling Yan, Feng Zhu, Hai Cheng, Zhenyu Li, Mingfeng Zhao, Kailin Xu

**Affiliations:** 1 Blood Diseases Institute, Xuzhou Medical University, Xuzhou, China; 2 Department of Hematology, The Affiliated Hospital of Xuzhou Medical University, No. 99 West Huaihai Road, Xuzhou, 221002 Jiangsu China; 3 Department of Hematology, Tianjin First Central Hospital, No. 24 Fu Kang Road, Tianjin, 300192 China

**Keywords:** B-cell acute lymphoblastic leukemia, Chimeric antigen receptor T-cell therapy, Granulocyte colony-stimulating factor, Cytokine release syndrome, Neurotoxic events, Efficacy

## Abstract

Few studies have reported the associations of granulocyte colony-stimulating factor (G-CSF) with cytokine release syndrome (CRS), neurotoxic events (NEs) and efficacy after chimeric antigen receptor (CAR) T-cell therapy for relapsed or refractory (R/R) B-cell acute lymphoblastic leukemia (B-ALL). We present a retrospective study of 67 patients with R/R B-ALL who received anti-CD19 CAR T-cell therapy, 41 (61.2%) patients received G-CSF (G-CSF group), while 26 (38.8%) did not (non-G-CSF group). Patients had similar duration of grade 3–4 neutropenia between the two groups. The incidences of CRS and NEs were higher in G-CSF group, while no differences in severity were found. Further stratified analysis showed that the incidence and severity of CRS were not associated with G-CSF administration in patients with low bone marrow (BM) tumor burden. None of the patients with low BM tumor burden developed NEs. However, there was a significant increase in the incidence of CRS after G-CSF administration in patients with high BM tumor burden. The duration of CRS in patients who used G-CSF was longer. There were no significant differences in response rates at 1 and 3 months after CAR T-cell infusion, as well as overall survival (OS) between the two groups. In conclusion, our results showed that G-CSF administration was not associated with the incidence or severity of CRS in patients with low BM tumor burden, but the incidence of CRS was higher after G-CSF administration in patients with high BM tumor burden. The duration of CRS was prolonged in G-CSF group. G-CSF administration was not associated with the efficacy of CAR T-cell therapy.

**Supplementary Information:**

The online version contains supplementary material available at 10.1007/s00262-024-03661-1.

## Introduction

Patients with relapsed or refractory (R/R) B-cell acute lymphoblastic leukemia (B-ALL) have an extremely poor prognosis. Chimeric antigen receptor (CAR) T-cell therapy targeting CD19 antigen has been demonstrated as an effective way to treat R/R B-ALL [1–4]. However, the associated toxicities, including cytokine release syndrome (CRS), neurotoxic events (NEs) and severe hemocytopenia, should be also evaluated in terms of predisposing risk factors and development of risk reduction strategies.

CRS is a systemic inflammatory response characterized by excessive release of various cytokines produced by CAR T–cells and other immune cells such as monocytes and macrophages [5, 6]. Pro-inflammatory cytokines, including C-reactive protein (CRP), interleukins (ILs) such as IL-1, IL-2 and IL-6, and granulocyte–macrophage colony-stimulating factor (GM-CSF), are among the most important mediators of CRS [5, 6]. NEs can occur after or before the onset of CRS, and the severity of NEs is associated with CRS [7, 8]. Activated CAR T–cells, endogenous immune cells and cytokines they released are all possible causes of NEs after CAR T-cell therapy [9–11]. IL-1 and IL-6 released by monocytes and macrophages are the main causes of CAR T-cell-associated CRS and NEs [12, 13]. Myeloid growth factors may play an important role on increasing the incidence and severity of CRS and NEs by promoting the secretion of pro-inflammatory cytokines during CAR T-cell therapy [12, 13]. Studies have shown that high serum concentrations of granulocyte colony-stimulating factor (G-CSF) and GM-CSF are associated with severe neurotoxicity [8, 12, 13]. Neutralization of GM-CSF can prevent CRS and neuroinflammation and do not inhibit CART19 cell function [14]. Another study showed that neutralization of GM-CSF or Talen-mediated knockout of GM-CSF in CAR T–cells could reduce the release of inflammatory cytokines from monocytes in vitro [15].

G-CSF has been used to promote the recovery of neutropenia and reduce the risk of infection [16]. It has been reported that patients with ALL and severe CRS are at a higher risk of infection after CD19 CAR T–cell therapy [17], and most infections occur during neutropenia. Most infections are caused by bacteria, followed by viral infections, fungal infections and so on [17–19]. Fried et al. [20] reported that 72% of R/R ALL or non-Hodgkin lymphoma patients treated with CD19 CAR T–cells developed severe neutropenia, with a median duration of 10 days. Due to the high incidence of neutropenia after CAR T-cell therapy [21–23], whether administration of G-CSF affect the toxicity and efficacy of CAR T-cell therapy deserves further study. Our previous study [24] showed that low-dose or short-term use of G-CSF in patients with multiple myeloma was not associated with the incidence or severity of CRS or NEs, and G-CSF administration did not affect the efficacy of CAR T-cell therapy. G-CSF administration did not affect the response rate and the incidence of CRS in patients with lymphoma, but might have an influence on the severity of CRS [25–28]. A recently study of patients with R/R B-ALL showed that early administration of G-CSF increased the incidence and prolonged the duration of CRS [29]. However, the association of G-CSF administration with the toxicities and efficacy of CAR T-cell therapy in R/R B-ALL remains unclear. In this study, we systematically analyzed the associations between G-CSF administration with CRS, NEs, infections and efficacy of CAR T-cell therapy in R/R B-ALL patients.

### Study design and patients

We retrospective analyzed patients with R/R B-ALL who received anti-CD19 CAR T-cell therapy at two clinical centers in China between June 2016 and November 2021. The clinical studies have been approved by the Ethics Committee and registered in the Chinese Clinical Trial Registry (ChiCTR-OIC-16008291, ChiCTR-ONN-16009862). Informed consent were obtained from all patients.

Prior to CAR T-cell infusion, enrolled patients were given lymphodepletion chemotherapy, which consisted of fludarabine (30 mg/m^2^/day, days -5 to -3) and cyclophosphamide (750 mg/m^2^, day -5). On day 0, patients received anti-CD19 CAR T-cells at the median dose of 2 × 10^6^ cells/kg (IQR 1.0–4.1 × 10^6^). The single-chain variable fragment (scFv) sequence specific for CD19 was derived from clone FMC63 and inserted in tandem with the human CD8 transmembrane, CD8 hinge, CD28 or 4-1BB costimulatory domain and CD3z intracellular regions as previously described [30, 31]. CARs targeting CD19 was synthesized and subcloned into lentivirus expression vector and stably expressed in CD3-positive T cells after transfection of lentiviral vector.

The indication of treatment with G-CSF was individually established per physician discretion, and it was administered at a dose of 1.25–5 μg/kg/d, according to the drug instruction. G-CSF administration was started when the neutrophil count was < 1.0 × 10^9^ /L and continued until the neutrophil count was ≥ 1.0 × 10^9^ /L for 2 days. Baseline clinical characteristics of patients, including age, gender, Philadelphia chromosome (Ph +) status, previous therapy lines, prior transplantation, extramedullary involvement and bone marrow (BM) tumor burden, were collected. Clinical manifestations and vital signs were recorded at any time during treatment. Neutrophil counts, concentration of Interleukin 6 (IL-6), ferritin and reactive protein C (CRP) during CAR T-cell therapy were also collected.

### Evaluation criteria for response and toxicity

Response assessment of patients was conducted according to the National Comprehensive Cancer Network guidelines [32]. CRS was graded according to the American Society for Transplantation and Cellular Therapy (ASTCT) Consensus Grading [33]. NEs and other adverse events (AEs) were evaluated according to the National Cancer Institute Common Terminology Criteria for Adverse Events v.4.03 [34].

### Statistical analysis

Data were presented as numbers (percentages) for categorical variables and median (interquartile range, IQR) for all continuous variables. Independent samples *t*-test and Mann–Whitney U test were used for continuous variables. Categorical variables were analyzed by Chi-squared or the Fisher exact test. Ordinal logistic regression was used to estimate risk factors for the occurrence of CRS. Event-free survival (EFS) was defined as time from the date of CAR T-cell infusion to the earliest occurrence of any of the following: failure to achieve response, death from any cause, relapse at any site, development of second malignant disease or the last follow-up. Overall survival (OS) was defined as time from the date of CAR T-cell infusion to death from any cause or the last follow-up. EFS and OS probabilities were estimated by the Kaplan–Meier method and were compared by the log-rank test. Both univariate and multivariable Cox regression analyses were applied to determine whether the G-CSF administration contributed to the long-term response. The variables included in the Cox models for multivariate analyses were those with *p* values < 0.1 in univariate analyses. The time of the last follow-up was January 31, 2023. SPSS Statistics 26.0 was applied for statistical analyses. *P* values less than 0.05 were considered statistically significant.

## Results

### Baseline characteristics

Seventy-eight patients with R/R B-ALL were enrolled, including 47 (60.3%) males and 31 (39.7%) females, with a median age of 26 years (IQR 14.75–41.25) (Supplementary Table 1). Eleven patients were excluded because they received treatment with G-CSF after successful treatment of CRS. Of the remaining 67 patients, 41 (61.2%) received G-CSF (G-CSF group), and 26 (38.8%) did not receive G-CSF (non-G-CSF group). The median percentage of BM blast cells was significantly higher in the G-CSF group than that in the non-G-CSF group (25%, IQR 5.0–71.5 versus 0.5%, IQR 0–13.5; *P* = 0.001). The proportion of male patients and the median neutrophil count at D0 were lower in G-CSF group than that in non-G-CSF group (*P* = 0.038 and < 0.001, respectively) (Table 1).

**Table 1:** Baseline characteristics of patients in G-CSF and non-G-CSF groups (*n* = 67)

	Total (*n* = 67)	G-CSF (*n* = 41)	Non-G-CSF (*n* = 26)	*P*
Age, y, median (IQR)	25(15–41)	26(15–41)	21.5 (13–40.75)	0.479
Male, *n* (%)	44(65.7)	23(56.1)	21 (80.8)	0.038
Ph + ALL, *n* (%)	14(20.9)	7(17.1)	7 (26.9)	0.334
Previous therapy lines, median (IQR)	4(2–6)	4(3–6)	3.5 (2–5.75)	0.168
Previous allo-HSCT, *n* (%)	14(20.9)	9(22.0)	5 (19.2)	0.790
Extramedullary involvement, *n* (%)	24(35.8)	11(26.8)	13 (50.0)	0.054
BM Blast Cell, %, median (IQR)	13(0–58.0)	25(5.0–71.5)	0.5 (0–13.5)	0.001
Absolute neutrophil count at D0,10^9^/L, median (IQR)	1.74(0.55–3.29)	0.68(0.155–2.0)	3.375 (1.99–4.225)	< 0.001

*G-CSF* Granulocyte colony-stimulating factor; *Ph+ALL* Philadelphia chromosome positive acute lymphoblastic leukemia; *IQR* interquartile range; allo-HSCT allogeneic hematopoietic stem cells transplantation; *BM* bone marrow

### Effect of G-CSF on neutropenia

Grade 3–4 neutropenia occurred in 70.1% (47/67) of the patients. The incidence of grade 3–4 neutropenia was 23.1% (6/26) in the non-G-CSF group. Grade 3–4 neutropenia occurred earlier in the G-CSF group with a median onset of day 0 (IQR -4.5–2.0) compared to day 5 (IQR 0.25–8.5) in the non-G-CSF group (*P* = 0.014) (Supplementary Table 2). The median minimum of neutrophil count in the G-CSF group and non-G-CSF group was 0.16 × 10^9^/L(IQR 0.025–0.635) and 0.295 × 10^9^/L(IQR 0.1525–0.63), without significant difference (Supplementary Table 2). The median duration of Grade 3–4 neutropenia in the two groups was 10.0 days (IQR 2.5–20.0) and 5.0 days (IQR 2.5–12.0), respectively, without significant difference (Supplementary Table 2).

### Association of G-CSF use with the incidence or severity of CRS and NEs

CRS occurred in 87.8% (36/41) of patients in G-CSF group and 65.4% (17/26) in non-G-CSF group, with 19.5% (8/41) and 7.7% (2/26) grade 3 or higher, respectively. 17.1% (7/41) patients in G-CSF group developed NEs, and 9.8% (4/41) had grade 3 or higher. None of the patients in non-G-CSF group developed NEs. The incidences of any grade CRS and NEs were higher in G-CSF group (*P* = 0.028 and 0.037, respectively) (Table 2); however, there was no difference in the incidence of severe (grade 3 or higher) cases (Table 2, Supplementary Fig. 1). Although there were no differences in baseline concentration of CRP, IL-6 and ferritin (*P* = 0.186, 0.457 and 0.630, respectively), the peak concentrations of these three markers were higher in G-CSF group (*P* = 0.007, 0.004 and 0.001, respectively) (Supplementary Fig. 2). Considering the difference in BM tumor burden between the two groups, which was an independent risk factor for CRS (OR 6.765, 95% CI [1.304–35.083], *P* = 0.023) (Supplementary Table 3) [35], we further divided the patients into two groups based on BM tumor burden (the median percentages of blast cells in BM, 13% as the cutoff). Stratified analysis showed that there were no differences in the incidence and severity of CRS between patients who used G-CSF and those did not in low-BM tumor burden group. None of the patients with low BM tumor burden developed NEs. However, there was a significant increase in the incidence of CRS for patients who used G-CSF than those did not in high-BM tumor burden group (*P* = 0.037), without significant differences in severity of CRS and incidence and severity of NEs (Table 2).

**Table 2:** Association of G-CSF with CRS or NEs (*n* = 67)

			All patients	G-CSF	Non-G-CSF	*P*
			*n* (%)			
All patients, *n*			67	41	26	
		CRS(Any Grade)	53(79.1)	36(87.8)	17(65.4)	0.028
		CRS(3–5 Grade)	10(14.9)	8(19.5)	2(7.7)	0.331
		NEs(Any Grade)	7(10.4)	7(17.1)	0	0.037
		NEs(3–4 Grade)	4(6.0)	4(9.8)	0	0.152
		Severe infections	32(47.8)	25(61.0)	7(26.9)	0.007
BM Blast cell, %						
	< 13, n		33	14	19	
		CRS(Any grade)	21(63.6)	9(64.3)	12(63.2)	1.000
		CRS(3–5 Grade)	1(3.0)	0	1(5.3)	1.000
		NEs(Any grade)	0	0	0	NA
		NEs(3–4 Grade)	0	0	0	NA
		Severe infections	8(24.2)	4(28.6)	4(21.1)	0.695
	≥ 13, *n*		34	27	7	
		CRS(Any grade)	32(94.1)	27(100)	5(71.4)	0.037
		CRS(3–5 grade)	9(26.5)	8(29.6)	1(14.3)	0.644
		NEs(Any grade)	7(20.6)	7(25.9)	0	0.300
		NEs(3–4 grade)	4(11.8)	4(14.8)	0	0.559
		Severe infections	24(70.6)	21(77.8)	3(42.9)	0.157

*G-CSF* Granulocyte colony-stimulating factor; *CRS* cytokine release syndrome; *NEs* chimeric antigen receptor T-cell-related neurotoxic events; *BM* bone marrow; *NA* not available

### Association of G-CSF with the onset time and duration of CRS

**Fig. 1:**
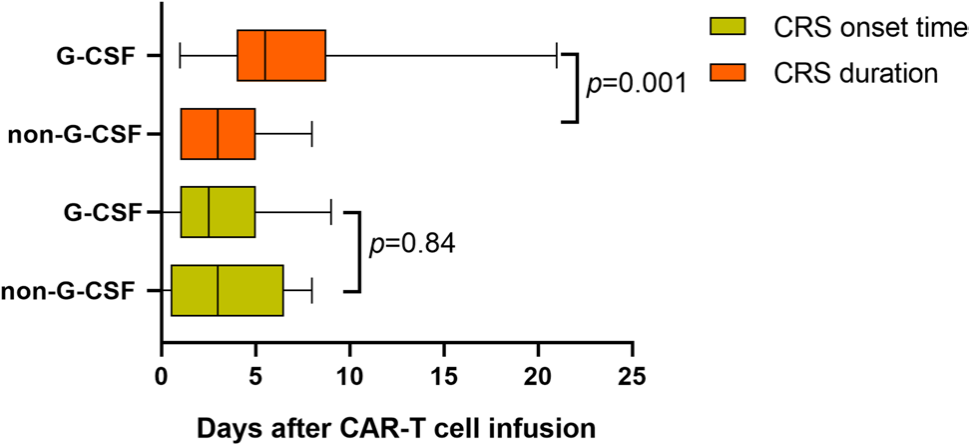
Comparison of CRS onset time and CRS duration of patients with CRS between those using G-CSF and those not using G-CSF groups. *G-CSF* Granulocyte colony-stimulating factor; *CRS* cytokine release syndrome; *CAR* chimeric antigen receptor

The median onset time of CRS in patients who used G-CSF and those who did not were on day 2.5 (IQR 1–5) and day 3 (IQR 0.5–6.5) after CAR T-cell infusion (*P* = 0.84), and the median duration were 5.5 days (IQR 4–8.75) and 3 days (IQR 1–5), respectively (*P* = 0.001) (Fig. 1).

### Association of G-CSF use with severe infections

Twenty-five patients (61.0%) and seven patients (26.9%) developed severe infections within 30 days post-CAR T-cell infusion in the G-CSF group and non-G-CSF group, respectively, including 14 upper respiratory tract infections, 13 pneumonias, 5 bacteremias, 1 intestinal infection and 1 skin soft tissue infection according to the infection site. In addition, according to the etiology of the infection, there were 18 bacterial infections, 11 viral infections, 2 fungal infections and 4 unknown infections. The incidence of severe infection was higher in patients using G-CSF (*P* = 0.007) (Table 2). Stratified analysis based on BM tumor burden (13% as the cutoff) showed that there were no differences in the incidence of severe infections between patients who used G-CSF and those did not in both low- and high-BM tumor burden groups (*P* = 0.695 and 0.157, respectively) (Table 2).There were no infection-related deaths in this study.

### Association of G-CSF use with the efficacy of CAR-T therapy

In G-CSF group and non-G-CSF group, 87.2% and 91.7% patients at 1 month, 82.1% and 95.7% patients at 3 months achieved complete remission or complete remission with incomplete count recovery (CR/CRi) after CAR T-cell infusion, respectively. There were no significant differences in CR/CRi rates between the two groups at 1 and 3 months after CAR T-cell therapy (Table 3). Stratified analysis based on BM tumor burden (13% as the cutoff) showed that there were no significant differences in CR/CRi rates between patients who used G-CSF and those did not in both low- and high-BM tumor burden groups (Table 3).

**Table 3:** Association of G-CSF with the efficacy of CAR-T therapy at 1 month (M1) and 3 months (M3)

	*n* (%)	ALL patients		*P*	BM Blast Cell(%) < 13		*P*	BM Blast Cell(%) ≥ 13		*P*
		G-CSF	Non-G-CSF		G-CSF use	G-CSF not use		G-CSF use	G-CSF not use	
		*n* = 41	*n* = 26		*n* = 14	*n* = 19		*n* = 27	*n* = 7	
At M1		*n* = 39	*n* = 24	0.699	*n* = 14	*n* = 18	0.295	*n* = 25	*n* = 6	0.488
	CR/CRi	34(87.2)	22(91.7)		11(78.6)	17(94.4)		23(92.0)	5(83.3)	
At M3		*n* = 39	*n* = 23	0.250	*n* = 14	*n* = 18	0.295	*n* = 25	*n* = 5	1.000
	CR/CRi	32(82.1)	22(95.7)		11(78.6)	17(94.4)		21(84.0)	5(100)	

Missing data at month 1: *n* = 4 (three patients died of toxicity before M1 and one patient had a follow-up shorter than 1 month).Missing data at month 3: *n* = 5 (three patients died of toxicity before M1, one patient had a follow-up shorter than 3 months, and one patient died due to disease progression before 3 months).*G-CSF* Granulocyte colony-stimulating factor; *ALL* acute lymphoblastic leukemia; *BM* bone marrow; *CR/CRi* complete remission or complete remission with incomplete count recovery.

The median EFS was 8.17 months (95% CI 4.94–11.40) in the G-CSF group and was not reached in the non-G-CSF group, and this difference was statistically significant (*P* = 0.009). We found no difference in OS between both groups: 11.70 months (95% CI 1.89–21.51) in the G-CSF and not reached in the non-G-CSF groups, respectively (Supplementary Fig. 3). Stratified analysis based on BM tumor burden (13% as the cutoff) showed that there were no significant differences in OS between patients who used G-CSF and those did not in both low- and high-BM tumor burden groups, as well as in EFS for patients with high BM tumor burden. However, EFS was higher in those patients who did not receive G-CSF in the low-BM tumor burden group (*P* = 0.041, Supplementary Fig. 4). To adjust for potential confounding factors, we constructed multivariate Cox models to test the proportional hazards assumption as well as the interaction terms with covariates in patients with low BM tumor burden. The multivariate analysis indicated that BM blast cells ≥ 0.17% (the median percentages of blast cells for patients with low BM tumor burden) was an independent negative prognostic factor for EFS in patients with low BM tumor burden (*P* = 0.04, 95% CI 1.058–12.004; Supplementary Table 4).

## Discussion

Previous study has shown that G-CSF treating chemotherapy-induced febrile neutropenia was associated with faster neutrophil recovery [36]. Cao, et al. [29] reported that early administration of G-CSF in CAR T-cell therapy did not reduce the incidence and duration of neutropenia. In this study, patients had similar minimum of neutrophil count and duration of grade 3–4 neutropenia in G-CSF group and non-G-CSF group. This may be related to the fact that fewer patients develop neutropenia in the non-G-CSF group.

CRS and NEs are the major toxicities of CAR T-cell therapy, which are related to the rapid and excessive release of multiple cytokines [37]. In vitro study has shown that a novel multi-cytokine inhibitor specifically inhibited pro-inflammatory cytokines without affecting the function of CAR T–cells [38]. Neutralization of cytokines may also be a potential strategy for managing these CAR-T-associated toxicities. Reducing infiltrating immune cells in the central nervous system leads to decreased levels of neuroinflammation and CRS [14]. G-CSF use is currently recommended for neutropenia in CAR T-cell therapy [34, 39, 40], but the safety remains controversial.

A retrospective study showed that prophylactic G-CSF was associated with an increased incidence of grade ≥ 2 CRS in lymphoma [27]. Gaut et al. [25] reported that patients treated with filgrastim had a similar incidence of CRS or immune effector cell-associated neurotoxicity syndrome (ICANS), but increased severity of CRS after CAR T-cell therapy in relapsed/refractory diffuse large B-cell lymphoma. In contrast, Lievin et al. [26] reported that early application of G-CSF did not affect the toxicity and efficacy of CAR T–cells. Barreto et al. [28] reported a similar incidence and severity of CRS and ICANS in patients treated with and without G-CSF in CAR T-cell therapy. However, there is no consensus on the risks and benefits of using G-CSF in CAR T-cell therapy in B-ALL.

In this study, patients in G-CSF group had higher incidence of CRS and NEs, higher peak concentrations of CRP, IL-6 and ferritin than that in non-G-CSF group. Further stratified analysis showed that G-CSF was not associated with the incidence or severity of CRS in patients with low BM tumor burden. However, there was a significant increase in the incidence of CRS for patients using G-CSF with high BM tumor burden, which is consistent with previous research [29]. Considering that only 7 patients with high BM tumor burden did not receive G-CSF, no further analysis was performed. It is necessary to expand the sample size to further clarify the association of G-CSF with CRS in patients with high BM tumor burden. In addition, we speculated that maybe an improved cytoreduction prior to CAR T–cell therapy could lower the BM leukemic burden, hence reducing the risk of G-CSF-induced CRS and NE, which makes G-CSF easier to use when indicated in severely neutropenic patients.

Our study also has limitations: This was a retrospective study, using G-CSF and the timing of G-CSF administration was at the discretion of physicians. NEs occurred in only 7 patients in G-CSF group in this study, so we did not further analyze the influencing factors of NEs. The sample size should be expanded to further improve this study.

In conclusion, G-CSF administration did not affect the efficacy of CAR T-cell therapy. Patients with low BM tumor burden using G-CSF appeared to be without association with the incidence or severity of CRS. However, the incidence of CRS was higher in patients with high BM tumor burden using G-CSF. The duration of CRS was prolonged in patients of G-CSF group than that in the non-G-CSF group.

### Supplementary Information

Below is the link to the electronic supplementary material.

Supplementary file1 (DOCX 770 KB)

Supplementary file1 (DOCX 14 KB)
