# Supplementary material for: Associations of granulocyte colony-stimulating factor with toxicities and efficacy of chimeric antigen receptor T-cell therapy in relapsed or refractory B-cell acute lymphoblastic leukemia
Source: Cancer Immunol Immunother. 2024 Apr 17;73(6):104. doi: 10.1007/s00262-024-03661-1 (PMC11024067; doi:10.1007/s00262-024-03661-1)
Supplement: Supplementary file 1 — Supplementary file1 (DOCX 770 KB) [file 262_2024_3661_MOESM1_ESM.docx]

**Supplementary Table 1 Characteristics of Patients (n=78)**

| **Variables** | **All patients** |
| --- | --- |
| **Gender, n (%)** |  |
| Male | 47(60.3) |
| Female | 31(39.7) |
| **Age, median (IQR)** | 26(14.75-41.25) |
| **Ph+ ALL, n (%)** |  |
| Yes | 15(19.2) |
| No | 63(80.8) |
| **Previous allo-HSCT, n (%)** |  |
| Yes | 16(20.5) |
| No | 62(79.5) |
| **Extramedullary involvement,** **n (%)** |  |
| Yes | 28(35.9) |
| No | 50(64.1) |
| **Target of CAR-T cell, n (%)** |  |
| CD19 | 78(100.0) |
| CD22 | 0 |
| **BM Blast Cell, %, median (IQR)** | 11(0-56.5) |

IQR interquartile range; Ph+ ALL Philadelphia chromosome positive acute lymphoblastic leukemia; allo-HSCT allogeneic hematopoietic stem cells transplantation; CAR chimeric antigen receptor

**Supplementary Table 2 Comparison of Grade 3-4 neutropenia onset time, minimum count and duration between G-CSF and non-G-CSF groups**

|  | All patients | G-CSF | non-G-CSF | ***P*** |
| --- | --- | --- | --- | --- |
|  | n=47 | n=41 | n=6 |  |
| Onset time of neutropenia (days), median (IQR) | 0(-4.0-3.0) | 0(-4.5-2.0) | 5.0(0.25-8.5) | 0.014 |
| minimum of neutrophil count(109/L), median (IQR) | 0.17(0.03-0.59) | 0.16(0.025-0.635) | 0.295(0.1525-0.63) | 0.666 |
| Duration of neutropenia (days), median (IQR) | 9.0(3.0-20.0) | 10.0(2.5-20.0) | 5.0(2.5-12.0) | 0.299 |

IQR interquartile range; G-CSF Granulocyte colony-stimulating factor

**Supplementary Table 3 Clinical factors associated with CRS (n=67)**

| **Variable** | **non-CRS(n=14)** | **CRS(n=53)** |  | **Univariate Analysis** |  | **Multivariable Analysis** | | |
| --- | --- | --- | --- | --- | --- | --- | --- | --- |
|  |  |  |  | ***P*** |  | **OR** | **95% CI** | ***P*** |
| Gender, n (%) |  |  |  | 0.261 |  |  |  |  |
| Male | 11(78.6) | 33(62.3) |  |  |  |  |  |  |
| Female | 3(21.4) | 20(37.7) |  |  |  |  |  |  |
| Age, years, n (%) |  |  |  | 0.508 |  |  |  |  |
| <25 | 8(57.1) | 25(47.2) |  |  |  |  |  |  |
| ≥25 | 6(42.9) | 28(52.8) |  |  |  |  |  |  |
| Previous allo-HSCT, n (%) |  |  |  | 0.134 |  |  |  |  |
| Yes | 5(35.7) | 9(17.0) |  |  |  |  |  |  |
| No | 9(64.3) | 44(83.0) |  |  |  |  |  |  |
| CNS involvement, n(%) |  |  |  | 0.644 |  |  |  |  |
| Yes | 4(28.6) | 12(22.6) |  |  |  |  |  |  |
| No | 10(71.4) | 41(77.4) |  |  |  |  |  |  |
| Extramedullary involvement, n (%) |  |  |  | 0.993 |  |  |  |  |
| Yes | 5(35.7) | 19(35.8) |  |  |  |  |  |  |
| No | 9(64.3) | 34(64.2) |  |  |  |  |  |  |
| Previous therapy lines, n (%) ^a^ |  |  |  | 0.707 |  |  |  |  |
| <4 | 5(35.7) | 23(43.4) |  |  |  |  |  |  |
| ≥4 | 8(57.1) | 29(54.7) |  |  |  |  |  |  |
| G-CSF, n (%) |  |  |  | 0.034 |  | 2.115 | 0.553-8.089 | 0.274 |
| Yes | 5(35.7) | 36(67.9) |  |  |  |  |  |  |
| No | 9(64.3) | 17(32.1) |  |  |  |  |  |  |
| CAR-T Cell Dose,  n (%) |  |  |  | 0.090 |  | 0.426 | 0.107-1.694 | 0.226 |
| <2*10^6^/kg | 4(28.6) | 29(54.7) |  |  |  |  |  |  |
| ≥2*10^6^/kg | 10(71.4) | 24(45.3) |  |  |  |  |  |  |
| BM Blast Cell, %, n (%) |  |  |  | 0.007 |  | 6.765 | 1.304-35.083 | 0.023 |
| <13 | 12(85.7) | 21(39.6) |  |  |  |  |  |  |
| ≥13 | 2(14.3) | 32(60.4) |  |  |  |  |  |  |

OR Odds ratio; 95%CI 95% confidence interval; IQR interquartile range; allo-HSCT allogeneic hematopoietic stem cells transplantation; CRS cytokine release syndrome; CNS central nervous system; G-CSF Granulocyte colony-stimulating factor; CAR chimeric antigen receptor; BM bone marrow.

^a^ the number of previous therapies lines in two patients was unknown

**Supplementary Table 4 Factors associated with EFS in patients with low BM tumor burden**

| **Variables(risk factor)** | **Univariable analysis** | | **Multivariable analysis** | |
| --- | --- | --- | --- | --- |
|  | ***HR* (95% *CI*)** | ***P*** | ***HR* (95% *CI*)** | ***P*** |
| Sex (male/female) | 1.338(0.435-4.115) | 0.612 |  |  |
| Age (<22/≥22years old) | 1.209(0.406-3.607) | 0.733 |  |  |
| Previous allo-HSCT (yes/no) | 0.612(0.135-2.766) | 0.523 |  |  |
| G-CSF use (yes/no) | 3.049(0.989-9.394) | 0.052 | 2.481(0.792-7.775) | 0.119 |
| CRS(yes/no) | 0.708(0.23-2.18) | 0.548 |  |  |
| BM Blast Cell(<0.17%/≥0.17%) | 4.147(1.253-13.723) | 0.02 | 3.564(1.058-12.004) | 0.04 |
| Previous therapy lines (<4/≥4) | 0.802(0.269-2.389) | 0.691 |  |  |
| CNS involvement(yes/no) | 1.815(0.608-5.416) | 0.285 |  |  |
| Extramedullary involvement(yes/no) |  | 0.961 |  |  |
| CAR-T Cell Dose (<2.6*10^6^/kg/≥2.6*10^6^/kg) | 0.646(0.21-1.984) | 0.445 |  |  |

EFS Event-free survival; HR hazard ratio; 95%CI 95% confidence interval; allo-HSCT allogeneic hematopoietic stem cells transplantation; G-CSF Granulocyte colony-stimulating factor; CRS cytokine release syndrome; BM bone marrow; CNS central nervous system; CAR chimeric antigen receptor


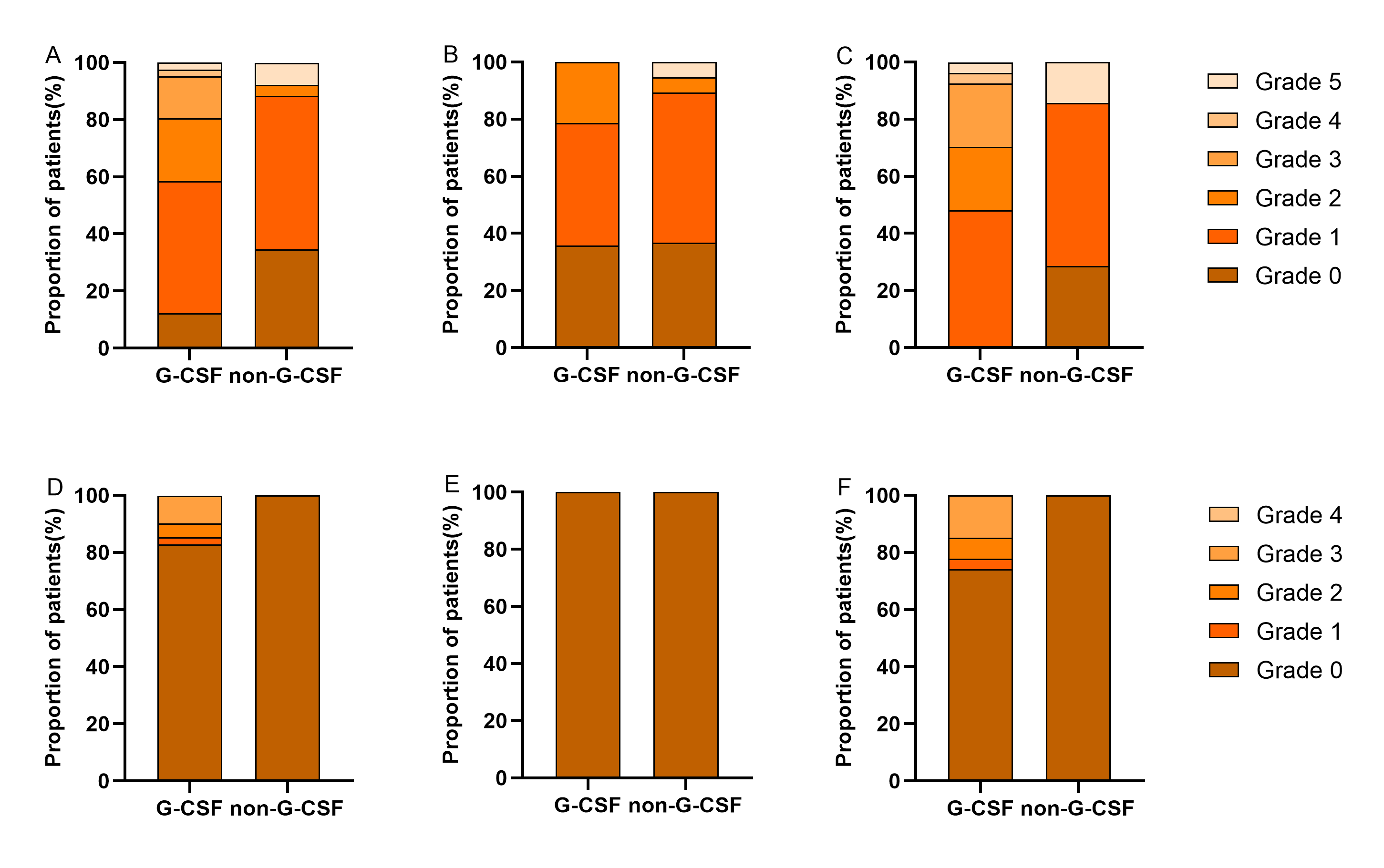


**Supplementary Fig. 1 Grades of CRS and NEs in G-CSF and non-G-CSF groups.** CRS grade (A). CRS grade of patients with BM blast cell < 13% (B) and ≥ 13% (C). NEs grade (D). NEs grade of patients with BM blast cell <13% (E) and ≥ 13% (F).

**
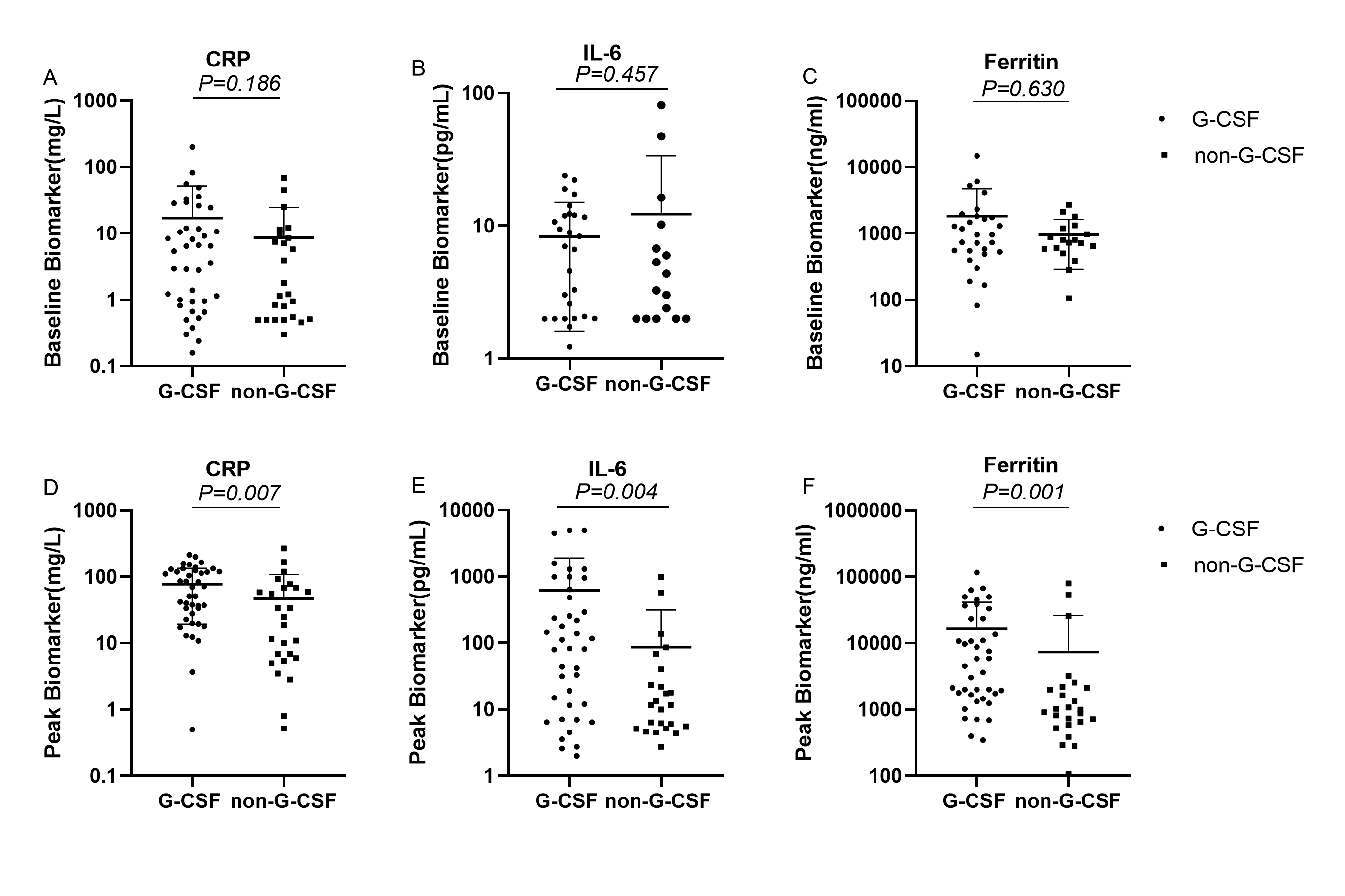
**

**Supplementary Fig. 2 Baseline and Peak concentration of CRP,IL-6 and Ferritin in G-CSF group and non-G-CSF group**. Baseline concentration of CRP (A), IL-6 (B) and ferritin (C). Peak concentration of CRP (D), IL-6 (E) and ferritin (F).

**
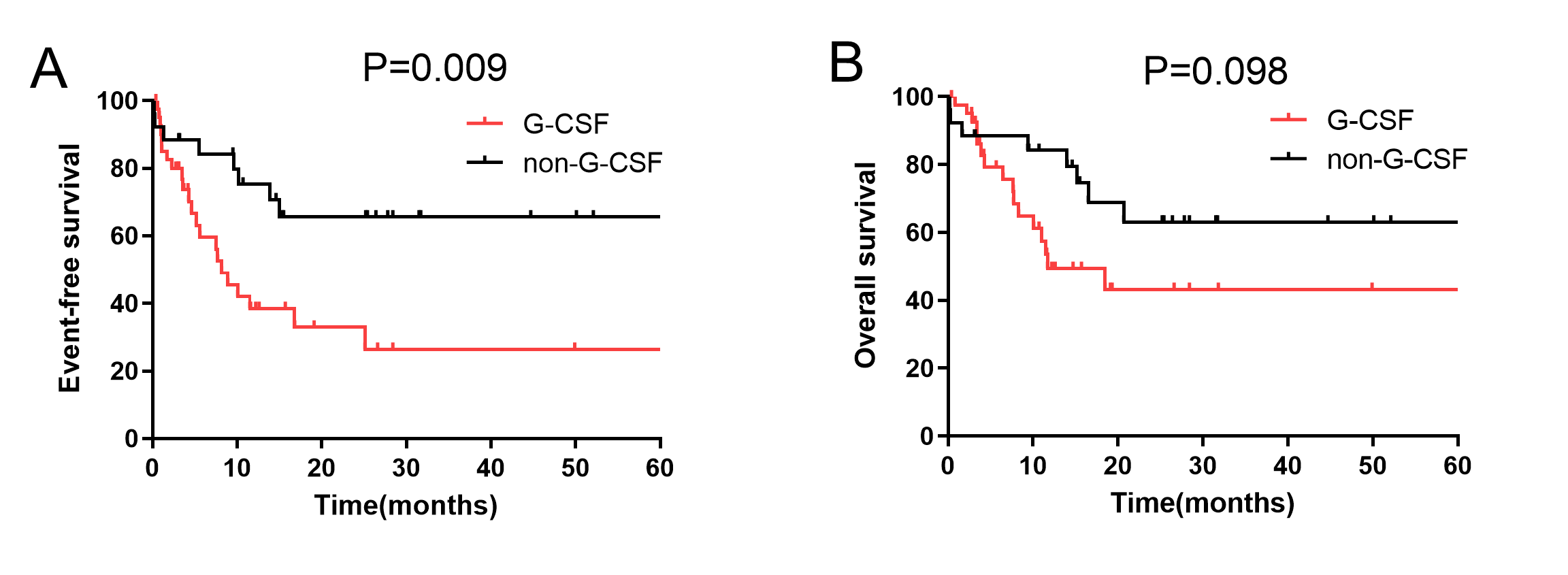
**

**Supplementary Fig. 3** **Event-free survival (EFS) and overall survival (OS) in G-CSF group and non-G-CSF group.** (A) Kaplan-Meier curves of EFS in G-CSF group and non-G-CSF group. (B) Kaplan-Meier curves of OS in G-CSF group and non-G-CSF group.

G-CSF Granulocyte colony-stimulating factor


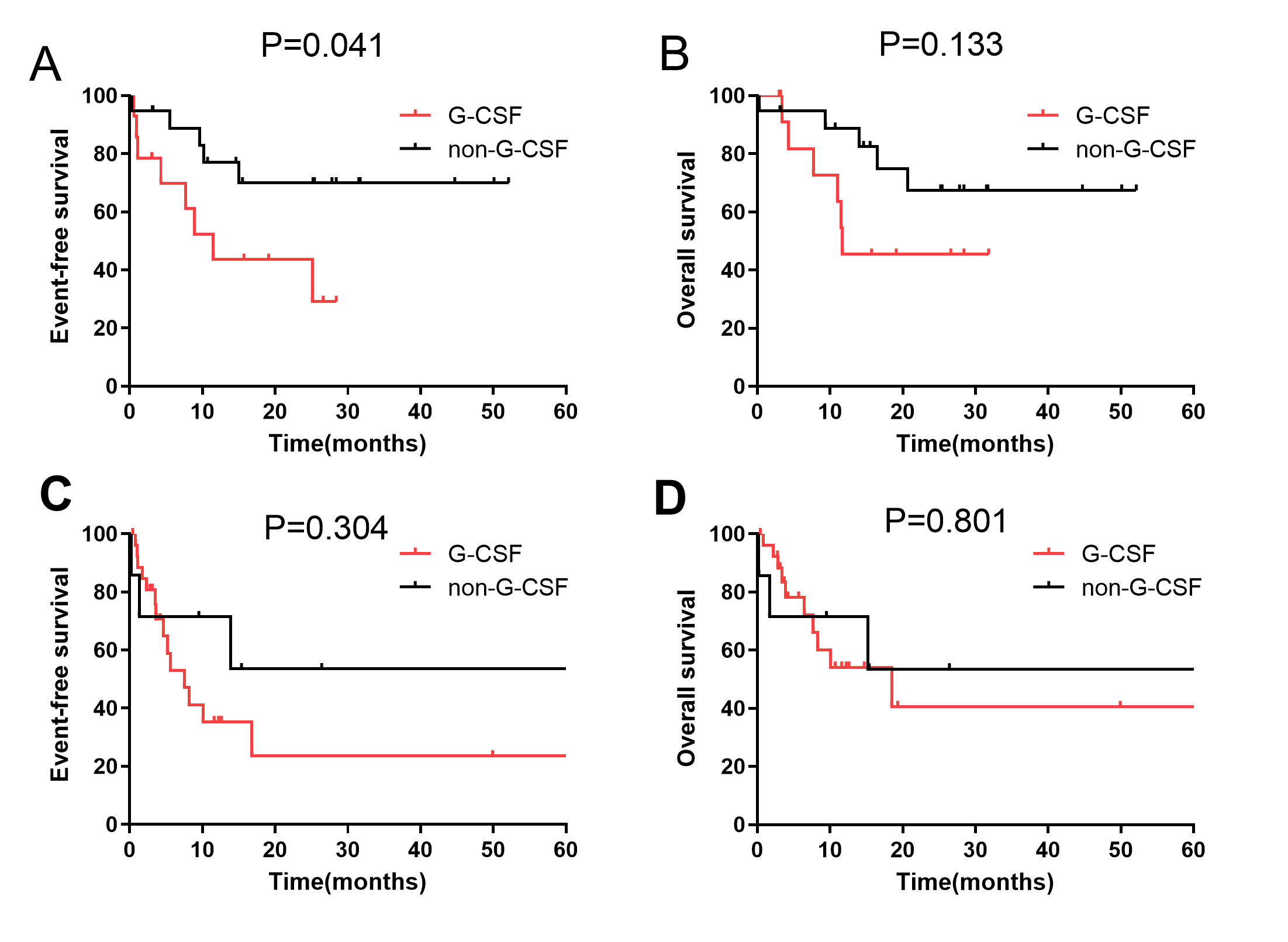
**Supplementary Fig 4 Event-free survival (EFS) and Overall survival (OS) in G-CSF group and non-G-CSF group** **of both patients with low and high BM tumor burden.** (A) Kaplan-Meier curves of EFS of patients with BM blast cell <13% in G-CSF group and non-G-CSF group. (B) Kaplan-Meier curves of OS of patients with BM blast cell <13% in G-CSF group and non-G-CSF group. (C) Kaplan-Meier curves of EFS of patients with BM blast cell ≥13% in G-CSF group and non-G-CSF group. (D) Kaplan-Meier curves of OS of patients with BM blast cell ≥13% in G-CSF group and non-G-CSF group.

G-CSF Granulocyte colony-stimulating factor; BM bone marrow
