# Supplementary material for: Associations of granulocyte colony-stimulating factor with toxicities and efficacy of chimeric antigen receptor T-cell therapy in relapsed or refractory B-cell acute lymphoblastic leukemia
Source: Cancer Immunol Immunother. 2024 Apr 17;73(6):104. doi: 10.1007/s00262-024-03661-1 (PMC11024067; doi:10.1007/s00262-024-03661-1)
Supplement: Supplementary file 2 — Supplementary file1 (DOCX 14 KB) [file 262_2024_3661_MOESM2_ESM.docx]

**Table 2 Comparison of Grade 3-4 neutropenia onset time, minimum count and duration between G-CSF and non-G-CSF groups**

|  | All patients | G-CSF | non-G-CSF | ***P*** |
| --- | --- | --- | --- | --- |
|  | n=47 | n=41 | n=6 |  |
| Onset time of neutropenia (days), median (IQR) | 0(-4.0-3.0) | 0(-4.5-2.0) | 5.0(0.25-8.5) | 0.014 |
| minimum of neutrophil count(109/L), median (IQR) | 0.17(0.03-0.59) | 0.16(0.025-0.635) | 0.295(0.1525-0.63) | 0.666 |
| Duration of neutropenia (days), median (IQR) | 9.0(3.0-20.0) | 10.0(2.5-20.0) | 5.0(2.5-12.0) | 0.299 |

IQR interquartile range; G-CSF Granulocyte colony-stimulating factor
